# What Happened to the Participants of the Math Olympiad 1971? A Multiple-Case Study Concerning the Occupational Success of the Winning Team from Hungary, Math Olympiad–Occupational Success

**DOI:** 10.3390/jintelligence7010009

**Published:** 2019-03-20

**Authors:** Benedikt Gasser

**Affiliations:** Swiss Registries and Data Linkage, University of Berne, 3012 Berne, Switzerland; benedikt.gasser@yahoo.com; Tel.: +41-788-170-711

**Keywords:** Math Olympiad, occupational success, occupational prestige

## Abstract

The aim of this multiple-case study was to find out how the most successful team of the 1971 Mathematical Olympiad in Hungary developed professionally. It is impressive that in 1971, no fewer than four participants on the Hungarian team received gold medals and four participants received silver medals. Seven of the eight analyzed participants on the team came from a grammar school in Budapest. Three of the four gold medal winners achieved remarkable academic positions. On average, professional positions were achieved (as scored by the magnitude prestige scale) well above the average for a normal grammar school sample. Interestingly, the calculated average of the Hungarian team (156.5 ± 15.5) was slightly higher than that of the comparison team from Sweden (142.8 ± 30.5), but this difference was not significant (p = 0.351). In principle, excellence seems to result in excellence. For several former participants on the Hungarian team, it was shown that they continued to be extremely successful in the field of mathematics, with a thematic focus in the field of statistics and probability calculations.

## 1. Introduction

“The only things you have are in your heart and brain" was a statement from Peter Frankl’s parents, who lived through the Holocaust and whose son showed impressive mathematical skills as a young boy [1]. Peter Frankl was later a participant on the most successful team of the International Mathematical Olympiad (IMO) in 1971. He attended high school in Budapest, later became a very successful mathematician, speaks many languages, Impressive person and is also an impressive personality in other areas of life.

The question remains: Why do mathematical abilities in childhood have such high prognostic validity for future occupational success? [2]. Recent evidence suggests that good mathematical skills are superior to other skills such as language and music in predicting future academic and occupational success [2,3,4,5,6], and it has been implied that good skills in mathematics may be crucial for the development of modern economies and the welfare of countries. The evidence comes from the educational economic debate, which places high importance on mathematics based on human capital and signal theory for success in labor markets [7,8,9,10]. Extrapolation analyses between educational achievements and economic growth in Germany implied that over the long term (over a lifespan from childhood until retirement), more than 13 trillion euros of additional gross domestic product (GDP) could be earned by matching the country’s educational achievements to those of leading European countries in the Program for International Student Assessment (PISA), such as Finland [8,10,11]. In particular, mathematical deficits appear to have a particularly negative effect on future labor market skills, and consequently a negative effect on welfare [10,11]. Recent scientific studies show a high association between educational achievements of the population (e.g., as measured by PISA) and long-term economic growth [8]. This can also be found in the school context by the findings of Schuler (2001), according to which math grades at the end of the ninth grade were identified as central predictors of a transition to initial vocational training and educational success as a conditional factor for further occupational success [6]. As result of the importance of other subjects, the empirical evidence of a negative impact of skill deficits is relatively comprehensive for mathematics compared to other subjects [4,12,13]. It was identified that in final college exams in Switzerland (where there is general access to university), 41% of written math examinations were insufficient; when the oral grade was added, 24% were still insufficient [5]. In Germany, an analysis of datasets from secondary school mathematics in Rheinland-Pfalz (MARKUS, about 1500 eighth-grade records) and primary school mathematics from the VERA study showed that student characteristics such as subject interest and effort had significant effects on grading [14]. Particularly, interest in mathematics had a positive effect [14].

It might be possible to decipher further hints when elucidating the occupational success of former participants in the Math Olympiad. It is possible that the prognostic capacity of the area of sounds fair highest ability differs only slightly according to the level of performance, because differences in occupational outcomes may not be connected to the difference between a slightly above average student and an excellent student. Furthermore, evidence-based conceptualizations quickly moved from viewing intellectually precocious individuals (including those who were mathematically gifted) as weak and emotionally labile to viewing them as highly effective and resilient individuals [15,16,17,18]. However, it was also indicated that intellectually precocious youths have strengths and weaknesses (which are also partly due to gender), and vast differences were revealed in their passion for different pursuits and their drive to achieve [15,16,17]. Deary et al. (2007) showed that for English pupils, based on an intelligence test administered at the age of 11 (Cognitive Ability Test, second edition (CAT2E) from Thorndike, Hagen & France, (1986)), performance on a countrywide final exam (General Certificate of Secondary Education (GCSE); General National Vocational Qualification (GNVQ)) at the age of sixteen (around five years later) is a valid predictor of future achievements—the latent correlation of the general factor of intelligence (g) with one of the authors’ constructed school g was r = 0.81 [19]. Concerning the relationship between intelligence (g) and 25 single subjects (n = 2720 to n = 68,125 depending on theme), values were highest for mathematics and varied between r = 0.77 (mathematics) and r = 0.43 (art and design). To summarize the key results, the relationship between general intelligence (g) and arts and music and sports was small compared to subjects with central-cognitive resources [19]. Furthermore, a study of mathematically-precocious youth (SMPY) cohorts showed that by their mid-thirties, these men and women appeared to be happy with their life choices and viewed themselves as equally successful (objective measures support these subjective impressions) [16]. Undoubtedly, based on current evidence, their outcomes should clearly be better than those of below-average students [20,21]. However, the question of how former absolute top-level mathematics students develop over the course of their lives is not elucidated. Are they also very successful in the labor market? Were they able to keep their narrow interest in mathematics during their lives? How is their outcome in comparison to other samples, such as other teams or academic cohorts? All of this leads to the central question of this study: How did former participants of the Math Olympiad develop professionally? How are they active on the labor market? What about their occupational success? As a hypothesis with potential falsification, it is postulated that the former participants do not differ in terms of occupational success from other samples.

## 2. Methods

### 2.1. Participants and Procedures

The 8 participants from the most successful team from Hungary in 1971 were analyzed concerning their occupational careers. The analyses were conducted for Péter Frankl (gold medal), Ferenc Göndöcs (gold medal), Péter Komjáth (gold medal), Imre Ruzsa (gold medal), Tamás Móri (silver medal), Ervin Bajmóczi (silver medal), Zoltán Füredi (silver medal), and András Nagy (silver medal). All of them attended colleges in Hungary; six of the eight attended college in Budapest, one was from Kaposvár, and one attended college in Győr.

### 2.2. Procedures and Statistical Analysis

The participants were analyzed via comprehensive social media research regarding their academic and occupational positions during their lives. Furthermore, scientific output such as papers and conference talks were analyzed. In order to quantify their occupational success, their current positions were scored. Occupational prestige was recorded by the magnitude prestige scale (MPS), a metric scale designed by Bernd Wegener for the former West Germany [22,23]. Occupational prestige has a mean of 63.8 and a standard deviation of 30.8. The scale ranges from a maximum of 186.8 for specialists or chief physicians to a minimum of 20 for assistants and unskilled manual workers [22,23]. A mathematician can reach 135.7 points, depending on their position (e.g., for a university professor, the MPS can go up to 167). In order to facilitate a comparison between former participants on the 1971 Hungarian team, the Swedish team was used as a comparison cohort. For this team, only occupational position was scored by MPS. It should be mentioned that two female participants were on the team.

For both teams, averages and standard deviations of MPS values according to current occupational position were recorded. In addition, the significance of the difference was calculated between the two teams using a 2-sided heteroscedastic T-test. Furthermore, another highly selective sample of former students (Cologne Gymnasiast Panel) was used in the results for another and broader sample.

## 3. Results

Looking at the lives of the former participants, various personalities with impressive lives are identifiable. Peter Frankl studied at Eötvös Loránd University under Gyula Katona, where he earned a doctorate [24,25,26]. In the 1980s he went to France and was active in science at the University of Paris [24,25,26]. Since 1988 he has lived in Japan and has published several works with Paul Erdős with a focus on extreme combinatorics [27]. Another gold medalist, Péter Komjáth, worked on combinatorial set theory and became a professor at Eötvös Loránd University [28]. Imre Ruzsa was also interested in combinatorics, number theory, and probability theory [29,30]. He studied at Eötvös Loránd University and in 1987 proved a theorem on the minimum number of elements in essential components, a notion of additive number theory [31,32]. An interest in combinatorics seems to have been shared by Zoltán Füredi as well [33]. Füredi also studied at Eötvös Loránd University, from which he graduated in 1978, with a focus on linear programming and hypergraphs. In 1981, he received a doctorate in Budapest from Gyula Katona, based on his studies of extremal hypergraphs and finite geometries [33]. He joined the Alfred Renyi Institute of the Hungarian Academy of Sciences in 1978, went to Rutgers University, became an assistant professor at the Massachusetts Institute of Technology (MIT) in 1986, and was an associate professor at MIT in 1990 and a professor of mathematics at the University of Illinois at Urbana-Champaign [33]. In 1989, Füredi, Imre Bárány, and László Lovász proved an asymptotic estimate of the number of planes dividing a set of S points in three-dimensional Euclidean space into two halves in general [33].

An interest in the field of statistics and probabilistic accounting by the former Hungarian team is expressed and supported by Tamás F. Móri, a professor in the Department of Probability and Statistics at Eötvös Loránd University [34]. His research focuses on pure mathematics, marginal theory with applications, combinatorial probability, and boundary theorems [34].

Other former members of the Hungarian team later had very productive years. Ervin Bajmóczi developed the topological Radon theorem [35]. Ferenc Göndöcs compiled a textbook on mathematical statistics that has been repeatedly published and is still used today [36]. András Nagy emigrated to Canada, where he does stem cell research. Interestingly, he went to Canada as a visiting scholar and stayed to study medicine. During his studies at the Lunenfeld-Tanenbaum Research Center at Mount Sinai Hospital in Toronto, he helped to develop the transformation of adult skin cells into embryonic stem cells [37,38]. Originally, Nagy earned a bachelor’s degree in mathematics (Eötvös Loránd University), and at the same time, he attended anatomy lessons because he did not like the idea of sounds fair spending his life with only a sheet of paper and a pencil [1]. This led to highly successful work as a biomedical researcher.

If one then attempts to grasp the professional positions or quantify the professional success, scoring can be carried out with the aid of the magnitude prestige scale (MPS) [22,23]. The Hungarian team scored as follows: Péter Frankl (gold medal) had an MPS score of 167 as a mathematician with a university assignment; Ferenc Göndöcs (gold medal), about whom there was the least information, has an MPS 135.4 as a mathematician; Péter Komjáth (gold medal) has an MPS 167 as a university professor; Imre Ruzsa (gold medalist), active as a mathematician, attained a score between general mathematician (MPS 135.4) and university professor (MPS 167); Tamás Móri (silver medalist) scored MPS 167 as a university professor; Ervin Bajmóczi (silver medalist) scored MPS 135 as a mathematician; Zoltán Füredi (silver medal) was also attributed the mean score; and Nagy András (silver medal), as a biomedical researcher, received the mean MPS of a university professor (167) and a doctor (186).

This means the entire Hungarian team had an average occupational prestige of 156.5 ± 15.5. As a comparison, reference can be made to the Swedish team. One person became a professor of physics, with an MPS score of 163.3, and one person became a medical doctor, with an MPS of 186.8 (both of these people were women); one person became an electrical engineer, with an MPS of 109.9; one person became a physical engineer, with MPS 109.9; one person became a university professor, with an MPS of 135.4; one person became a mathematics lecturer with a university position. Here, similar to the Hungarian team, the average MPS is 135.4 and 167 [11,22,23]. This yields an average prestige of 142.8 ± 30.5 according to Wegener’s magnitude prestige scale.

Interestingly, the average of the Hungarian team, calculated at 156.5 ± 15.5, was slightly higher than that of the Swedish team, at 142.8 ± 30.5, but the difference between the two samples was not significant (p = 0.351).

Compared to an analyzed sample of German panel data of a cohort of former high school students, the Cologne Gymnasiast Panel, a sample covering the long period between the tenth year of school and labor and life participation, with surveys at ages 30, 43, and 56 years, higher values were achieved by former IMO participants. For the analyzed subsample, for a normal student career with a sequence of high school, college, university, and work, there are lower values at the age of 30 years with 109.6 ± 37.4, 43 years with 120.2 ± 36.1, and 56 years with 122.7 ± 35.4, compared to the IMO participants. Interestingly, average intelligence was also above average for this sample, at around 111, compared to normal populations [39,40,41,42,43].

## 4. Discussion

The aim of this study was to determine how former Math Olympiad participants have developed over their lives. It was postulated that they would not differ from normal college students in terms of occupational success. This hypothesis seems falsifiable. Based on the analysis of the most successful team in the Math Olympiad of 1971 from Hungary, it can be shown that outstanding mathematical ability in youth leads to higher occupational positions. The first hints at the validity of these results were derived from Terman (1954). Since a very high intelligence quotient can be assumed for the analyzed group of participants at IMO, the above statement is also compatible with findings from intelligence research, according to which intelligence has a very high long-term prognostic validity for occupational success [19,44,45]. What is astonishing and not shown by comparable analysis from Lubinski and colleagues, or from the Lothian birth cohort, is that everyone on the Hungarian team except András Nagy stayed in the field of mathematics their whole life [15,16,17]. These participants did not even change their general interest during this long period (around 50 years), which implies some sort of high personal stability in different fields of development, probably up to moral thinking [32,33,46,47]. Interestingly, no women were on the team. This is partly in line with the SMPY cohorts investigated by Lubinski & Benbow (2006), indicating that more mathematically-precocious men than women enter math and science careers without necessarily a loss of talent, because women secure a similar proportion of advanced degrees and high-level careers in areas corresponding more with the multidimensionality of their ability-preference pattern (e.g., administration, law, medicine, and social sciences) [16]. This would be in accordance with the findings for the Swedish team, with the two female participants having the highest MPS values. However, at IMO in 1971, there were only five female participants in total (two from Sweden, one from France, one from Mongolia, and one from Czechoslovakia), implying some sort of gender drift toward males [39,40,48,49,50,51].

By their mid-thirties, the men and women from the SMPY cohorts appeared to be happy with their life choices and viewed themselves as equally successful; objective measures support these subjective impressions with accomplishments such as tenure at a major research university, top executives at name-brand or Fortune 500 companies, attorneys at major firms or organizations, published books and refereed articles, secured patents, and millions amassed in grants [35,36,37]. Trying to compare findings from IMO with PISA, it becomes evident that, for example, Greece has a very good IMO ranking but only a modest PISA ranking [52,53]. Switzerland is the best in Europe, according to PISA, but has a modest position in IMO, and the same pattern can be identified for other countries (e.g., Estonia), allowing us to suggest that a country’s success at IMO is not that dependable from the general educational system [52,53].

Undoubtedly, the study has some limitations. On the one hand, the scoring of occupational prestige can cause problems. A mathematician’s 135.7 points, for example, compared to a chief physician’s 188, is a relatively low value. In addition, there is a tracking problem. Although individual proof was available for some people, information on others was only rudimentary, consequently affecting the validity of results.

In summary, the relevance of mathematics grades as a condition for professional success was repeatedly demonstrated [2]. The empirical findings can be relatively well-embedded in the current evidence. Positive effects of mathematics grades were repeatedly shown, for example with regard to later income, often associated with better education [2,54,55,56]. The recent empirical findings that even young people with excellent mathematical skills are very successful in their careers seem to further substantiate the relevance of the subject and can be classified accordingly in the most recent discussion on education economics [8,10,11]. The analysis reveals, however, that in the vast majority of cases, outstanding ability in mathematics seems to manifest in far above average to outstanding professional positions. Despite the complexity of tracking and analyzing curricula over such a long lifespan, it is impressive that, on average, the people in this study reached occupational positions that are far above the average of normal samples (for example, in a comparison of the sample of the Cologne college school panel selected as one control cohort). Keep in mind that this cohort also had an average intelligence quotient of 111, which is highly above the normal population, making this sample highly selective [39,40,41,42,43]. The well-known phenomenon that educational successes are reproduced also seems to apply to the participants of the Math Olympiad, and consequently social inequalities are transformed in later life [20,21,39,40,41,42]. Outstanding mathematical skills were transformed into other areas of life, and for the analyzed team this ranged from pure mathematics to medicine to brilliant language comprehension or even coordinative skills. Young participants in a Math Olympiad thus seem to develop superbly in principle, although exceptions exist [11].
